# Altered expression of the *DISC1* gene in peripheral blood of patients with schizophrenia

**DOI:** 10.1186/s12881-020-01132-9

**Published:** 2020-10-02

**Authors:** Xiaoqian Fu, Guofu Zhang, Yansong Liu, Ling Zhang, Fuquan Zhang, Conghua Zhou

**Affiliations:** 1 Department of Clinical Psychology, Suzhou Guangji Hospital, The Affiliated Guangji Hospital of Soochow University, Suzhou, Jiangsu China; 2 Wuxi Mental Health Center of Nanjing Medical University, 156 Qianrong Road, Wuxi, China; 3 Department of Psychiatry, The Affiliated Brain Hospital of Nanjing Medical University, 264 Guangzhou Road, Nanjing, Jiangsu Province China; 4 School of Computer Science and Telecommunication Engineering, Jiangsu University, Zhenjiang, China

**Keywords:** Schizophrenia, *DISC1*, Antipsychotics

## Abstract

**Background:**

Schizophrenia is a severe, heritable, and refractory psychiatric disorder. Several studies have shown that the disrupted in schizophrenia 1 (*DISC1*) gene is closely associated with schizophrenia by its role in neuronal morphology, synaptic function, brain development, and dopamine homeostasis etc. This study intended to investigate the expression levels of *DISC1* gene in schizophrenia patients compared with healthy controls, and the expression variation of *DISC1* gene before and after antipsychotic treatment in schizophrenia patients.

**Methods:**

In this study, we compared *DISC1* expression levels in blood of 48 healthy controls, and 32 schizophrenia patients before and after 12 weeks of antipsychotic treatment using real-time quantitative PCR (RT-qPCR) analysis.

**Results:**

The expression levels of *DISC1* gene in peripheral blood mononuclear cells of schizophrenia patients before antipsychotic treatment were higher than those in healthy controls (*P* < 0.01); whereas after antipsychotic treatment, the expression levels of *DISC1* gene in peripheral blood mononuclear cells of schizophrenia patients still remained increased (*P* < 0.01).

**Conclusions:**

Our study provided further support for the involvement of *DISC1* in the development of schizophrenia.

## Background

Schizophrenia (SCZ) is a chronic, severe mental disorder, accompanied by positive symptoms such as hallucinations, delusions, and negative symptoms including decreased motivation, anhedonia, cognitive impairment and social dysfunction [1–3]. The etiology of SCZ remains unclear, with environmental and genetic factors thought to play an important role [4, 5].

Since the disrupted in schizophrenia 1 (*DISC1*) gene was first discovered in a Scottish family with an unusually high incidence of SCZ and other mental disorders [6–8], it has been identified as a candidate risk gene for SCZ in multiple genetic and clinical association studies [6, 9]. *DISC1* is a regulator of glutamate function, whose transmission dysfunction is considered to be at the core of mental disorder pathology [10, 11]. Devine et al. proposed that *DISC1* controls transport of a wide range of neuronal cargos, including neurotransmitter receptors, mRNAs, vesicles, and mitochondria and regulates neuronal morphology and synaptic function, making it a key factor in the regulation of neuronal intracellular trade [12]. Degradation of the *DISC1* subtype has been shown to lead to neurodevelopmental abnormalities, suggesting that the breakdown of *DISC1* disrupts the mitochondrial dynamics of axons and dendrites [13].

Prenatal brain development has been implicated in the risk of mental illness, while gray matter has been shown to be substantially decreased in the neonatal homozygous for the *DISC1* rs821616 serine alleles [14]. *DISC1* has also been found to regulate astrocytes via modulating RAS/MEK/ERK signaling mediated by RASSF7 in the embryonic brain, whose defects might contribute to SCZ [7]. In addition, *DISC1* translocation has been associated with decreased white matter integrity in the frontal junction and associated fiber bundles in both animal models and patients with psychosis [14, 15]. This cortical thinning observed in individuals with *DISC1* translocation was confirmed to be highly similar to SCZ [16]. The *DISC1* and *SLC12A2* genes have been identified as SCZ risk genes, and their role in GABA depolarization co-regulates the development of hippocampal neurons. Two SNPs (rs1000731 in *DISC1* and rs10089 in *SLC12A2*) have been shown to increase the risk of SCZ interactively, with subjects carrying both SNPs displaying a significant reduction in hippocampal activation as well as reduced connectivity with the prefrontal cortex [17]. In a model organism study, *DISC1* has been shown to play a role in sleep regulation, suggesting a possible association between *DISC1* and SCZ in terms of sleep [18]. Abnormal *DISC1* and NDEL expression is linked to impaired cognitive function, which is a major symptom of SCZ [19], furthermore, it has been hypothesized that a relationship exists between *DISC1* and dopamine in SCZ [5], as dopamine homeostasis is closely related to the integrity and expression level of *DISC1* [20].

The use of antipsychotics by patients with SCZ has been known to alter gene expression in some cases, metabolism-related genes are an example of aforementioned genes whose expression has been shown to be affected [21, 22]. Moreover, several studies have revealed that many genes were up or down regulated in SCZ patients, while the gene expression of some genes may be restored to normal levels after treatment with antipsychotics [21, 23].

Although the effects of antipsychotic drugs on gene expression have been well studied, reports focusing on *DISC1* are scarce and often conflicting. A typical example of such contrasting reports is a study that found *DISC1* expression to be increased and remain elevated in peripheral blood mononuclear cells (PBMCs) of SCZ patients of Sinhalese descent, despite the use of antipsychotics [23]. From the above studies, we know that antipsychotics can affect gene expression and treat SCZ on a molecular level [24], and additionally that *DISC1* is strongly associated with SCZ. It is therefore necessary to investigate the impact of antipsychotic treatment on the expression of *DISC1*. As far as we know this is the first study of its kind in the Chinese Han population and aims to discover the therapeutic significance of *DISC1* in SCZ.

## Methods

### Ethics statement

The clinical research procedures were confirmed by the ethics committee of the Wuxi Mental Health Center and followed the World Medical Association Declaration of Helsinki-Ethical Principles for Medical Research Involving Human Subjects. All enrolled participants (or their legal guardian in cases where the patient lacked the capability to provide consent) were required to sign an informed consent form when the patient was assessed by a psychiatrist.

### Subject recruitment

All participants were inducted from the Han Chinese population in Shanxi province of China, including 32 SCZ patients and 48 healthy controls (HCs). There was no significant statistical difference in gender, age and ethnicity between the SCZ group and the HC group. (Table 1).

**Table 1:** Demographics of HC subjects and SCZ patients

Variable	HC	SZ	t/χ2	*P* values
Gender (M/F)	17/31	14/18	0.56	0.45
Age	31.56 ± 6.88	35.84 ± 12.05	−1.89	0.07
Ethnicity	Han	Han		
Age at onset		26.28 ± 10.47		
Duration of illness (month)		130.57 ± 106.71		
PANSS score		102.16 ± 14.11		

Notes: *HC* Healthy control; *SZ* Schizophrenia patients before antipsychotics; Abbreviation: *M* Male; *F* Female

The SCZ patients were recruited from the First Hospital of Shanxi Medical University and diagnosed by two experienced psychiatrists based on the Diagnostic and Statistical Manual of Mental Disorders, Fourth Edition (DSM-IV) criteria. All SCZ patients were antipsychotic-naïve and received a 12-week course of antipsychotic treatment after commencement of the study. The SCZ patients were treated with the oral second-generation antipsychotics which were comprised of olanzapine (*n* = 10), quetiapine (*n* = 6), aripiprazole (n = 6), risperidone (*n* = 5), amisulpride (*n* = 3) and ziprasidone (*n* = 2). All patients in the SCZ group showed improved clinical symptoms with a reduction rate of over 25% according to the evaluation of the Positive and Negative syndrome Scale. The following diseases such as serious organic brain injury, alcohol or substance abuse, epilepsy, intellectual disability and other mental disorders should be excluded. On the basis of a Structured Clinical Interview for DSM-IV and Non-patients edition, the HC participants were randomly enrolled from local communities of Shanxi Province and there were no mental or neurological disorders among them.

### Analysis of gene expression by real-time quantitative polymerase chain reaction (RT-qPCR)

RT-qPCR was utilized to analyze the expression levels of *DISC1* in PBMCs of 48 HCs and 32 SCZ patients before and after the 12-week antipsychotic treatment, as described previously [25]. AGGATGAGGAGGAGGAGAGC (forward) and TTTGGGCATTTTCCATTCAT (reverse) were the PCR primers for *DISC1*. Prior to this total RNA was extracted from PBMCs using TRIzol reagent (Invitrogen, USA) with on-column DNase I treatment as described by the manufacturer.

### Statistical analysis

SPSS 20.0 was used for all statistical analysis. The comparative Ct (2^−ΔΔCt^) method was used to analyze the relative expression level of *DISC1* of each individual after normalization to the glyceraldehyde 3-phosphate dehydrogenase (GAPDH) gene. The Mann-Whitney U test was used to compare the expression levels of *DISC1* in SCZ patients before and after the 12-week antipsychotic treatment, as well as the HC subjects [26]. The threshold for statistical significance was set at *P* < 0.01 (two-tailed).

## Results

**Fig. 1:**
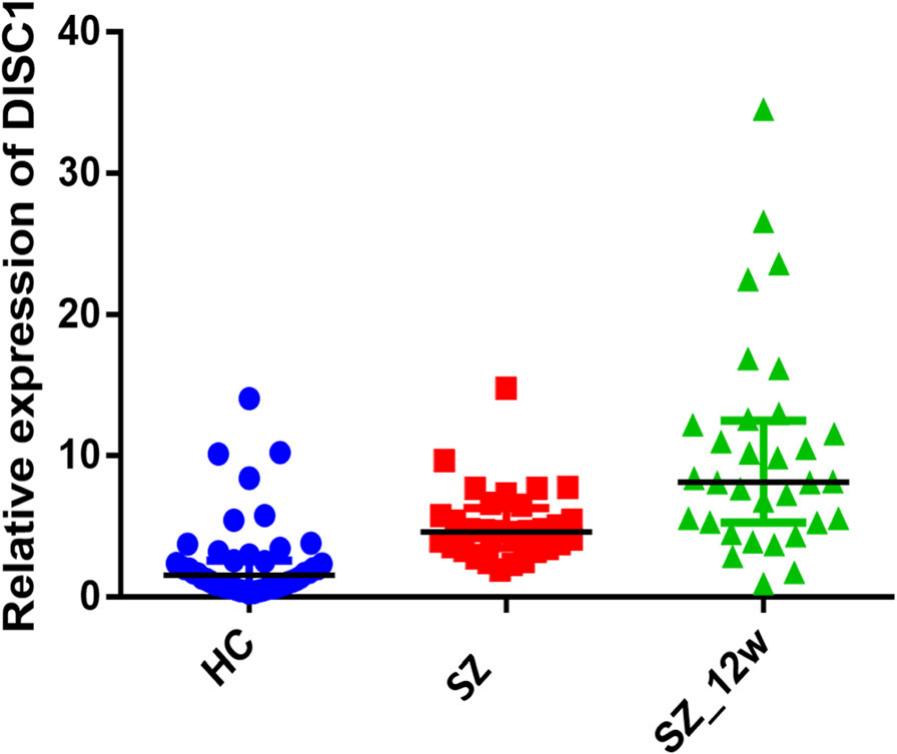
Comparison of *DISC1* expression levels in SZ, SZ_12w and with those in HCs. Notes: HCs: Healthy controls; SZ: schizophrenia patients before antipsychotics; SZ_12w: schizophrenia patients after 12-week antipsychotics; ① The expression levels of *DISC1* gene in PBMCs of SCZ patients before antipsychotics were higher than those in HC subjects. *P* < 0.01 (Mann Whitney U test). ② The expression levels of *DISC1* gene in PBMCs of SZ_12w patients were higher than those in baseline SZ patients. *P* < 0.01 (Mann Whitney U test)

The expression levels of *DISC1* gene in PBMCs of SCZ patients before antipsychotics were higher than those in HC subjects (Z = 5.34, *P* < 0.01). Nevertheless, the expression levels of *DISC1* gene in PBMCs of SCZ patients were still elevated after 12-week antipsychotic therapy (Z = 3.59, *P* < 0.01) (Table 2 and Fig. 1).

**Table 2:** Comparison of median *DISC1* levels in SZ, SZ_12w and with those in HCs

Group	Median (IQR)	Z values	*P* values
HCs (*n* = 48)	1.54 (0.71–2.58)		
SZ (*n* = 32)	4.62 (3.48–6.31)	5.34①	< 0.01
SZ_12w (*n* = 32)	8.15 (5.28–12.48)	3.59②	< 0.01

Notes: *HCs* Healthy controls; *SZ* Schizophrenia patients before antipsychotics; SZ_12w: schizophrenia patients after 12-week antipsychotics; IQR: inter-quartile range; ① HC Vs SZ; ② SZ Vs SZ_12w

## Discussion

This study found that the expression levels of *DISC1* gene in PBMCs of untreated SCZ patients were higher than those in HC subjects and continued to elevate despite 12 weeks of antipsychotic treatment. Our findings were consistent with previous research which reported that *DISC1* expression increased in PBMCs of antipsychotic-naïve SCZ patients when compared to HCs and remained increased despite six to 8 weeks of antipsychotic treatment [23] (Table 3).

**Table 3:** The prior evidence for *DISC1* expression between SCZ patients and HCs

Study	Sample size	Tissue	*DISC1* expression in SCZ patients.	Consistent with our results
[23]	10 SCZ patients and 11 HCs	PBMCs	increased	Yes
[29]	43 SCZ patients and 79 HCs	postmortem brain tissue	increased	Yes
[19]	69 SCZ patients and 63 HCs	whole blood	decreased	No
[35]	family (117 probands) and case-control (210 pairs)	postmortem brain tissue	unchanged	No

Notes: *SCZ* Schizophrenia; *HCs* Healthy controls; *PBMCs* Peripheral blood mononuclear cells; *DISC1* Disrupted in schizophrenia 1

PBMCs are routinely employed in investigating gene expression as a substitute to brain tissue [27] because of its similar gene expression profile to brain tissue and relative ease of access [23, 28]*. DISC1* protein expression in hippocampal tissue has been reported to be elevated in the patients with SCZ [29] (Table 3). Our results indicated that *DISC1* expression levels in PBMCs of SCZ patients may not respond to drug therapy, which supports *DISC1* as a trait-related rather than a state-related biomarker for SCZ. There is also the possibility that the effects of drug therapy on *DISC1* expression may be specific to specific tissues or organs [19], therefore the effects of antipsychotics on *DISC1* expression in other tissues should be further studied.

An animal model of SCZ, Disc1-l100p mice, display several SCZ-like symptoms such as hyperactivity, abnormal pre-pulse inhibition, enlarged lateral ventricles, decreased social activity [4, 30] and tend to have a prolonged release of dopamine, which is consistent with clinical findings that increased release of synaptic dopamine in the striatum of SCZ patients can lead to the deterioration of psychiatric symptoms [5, 31]. Antipsychotics could improve the behavior abnormalities and break the psycho-stimulatory effect of amphetamine in Disc1-l100p mutants [3, 5]. Su et al. discovered that the levels of the D2R-DISC1 complex were elevated with reduced GSK-3α/β (Ser21/9) phosphorylation in post-mortem brain tissue of patients with SCZ and disc1-l100p mutant mice, while interfering peptides that disrupt the D2R-DISC1 complex and haloperidol can potentially reverse behaviors associated with SCZ [3]. They further hypothesized that *DISC1* facilitated the D2-receptor-mediated transmission of GSK-3 signals, which could be responsible for SCZ’s psychotic symptoms [32] through D2R-DISC1 interaction. Hippocampal neurons of DISC1-deficient mice displayed exaggerated endoplasmic reticulum calcium responses that led to hyperactive dopamine function, while antipsychotic drugs such as clozapine and haloperidol, were found to be capable of reversing the abnormal endoplasmic reticulum calcium dynamics caused by *DISC1* dysfunction [12].

These studies mentioned above have indicated that different types of *DISC1* mutation or dysfunction are related to hyperactive dopamine function or the maturation of dopamine neurons, while some model organism studies showed that antipsychotics or antipsychotic substances could reverse dopamine-related dysfunction. The pathogenesis of SCZ is currently unknown but a popular hypothesis is that SCZ is caused by dopamine dysfunction, as most antipsychotics block dopamine receptors [33, 34]. Due to the relationship between *DISC1* and dopamine we concluded that it is necessary to study the effects of antipsychotics on *DISC1* expression in humans our study did not, for the time being, identify changes in *DISC1* expression that were consistent with the effectiveness of antipsychotics and improvement in patient symptoms. However, the possibility that *DISC1* expression response to drug therapy may be delayed does exist and needs to be verified by increasing the follow-up time in the future.

Several groups have investigated the expression levels of *DISC1* in SCZ patients compared to HCs. Fazio et al. [19] found decreased expression levels of *DISC1* in whole blood of SCZ patients, which may be due to different RNA sources used, since they extracted the RNA from whole blood instead PBMCs. There was also study that failed to find a difference in expression of *DISC1* between SCZ patients and HCs in the dorsolateral prefrontal cortex from postmortem brain tissue [35] (Table 3). The aforementioned inconsistencies can be explained by the fact that *DISC1* gene might express differently in diverse tissues or brain regions of the body; heterogeneity of study samples could lead to variation in *DISC1* expression [19]; and/or insufficient statistical power due to the small sample size. In consequence, due to the small sample size of our current study, we still need to expand the sample size in the future to further verify our results and the expression levels of *DISC1* in different tissues, organs or lineages warrant further study.

The main restriction of this study was relatively small sample size, which might influence the statistical effects for comparing *DISC1* expression level between SCZ patients and HC subjects, a larger sample may be required to validate the present findings in the future. Secondly, for the qPCR experiments, we used one control gene (*GAPDH*) for normalization, and it is therefore possible that changes in *GAPDH* rather than *DISC1* explain the results. Although *GAPDH* is a common control gene for normalization for qPCR analysis and was used alone as a control gene for normalization in many literatures, it still needs to additionally measure (and correct *DISC1* expression by) at least one another control gene to show that this is not the case in the future. At last, the relationship between clinical symptoms and expression data was lacking.

## Conclusions

Our results supported the involvement of the *DISC1* gene in the development of SCZ.

## Data Availability

For access to the data in this paper, interested researchers may contact the corresponding author.

## Abbreviations

*DISC1*: Disrupted in schizophrenia 1
DSM-IV: Diagnostic and statistical manual of mental disorders, 4th edition
*GAPDH*: Glyceraldehyde-3-phosphate dehydrogenase
HCs: Healthy controls
IQR: Interquartile range
PBMCs: Peripheral blood mononuclear cells
RT-qPCR: Real-time quantitative polymerase chain reaction
SCZ: Schizophrenia
SZ: Schizophrenia patients before antipsychotics
SZ_12w: Schizophrenia patients after 12-week antipsychotics
